# Effect of bleaching agents on enamel surface of bovine teeth: A SEM study

**DOI:** 10.4317/jced.53011

**Published:** 2017-01-01

**Authors:** Ana-Cristina Pimenta-Dutra, Rodrigo-de Castro Albuquerque, Luís-Fernando-dos Santos-Alves Morgan, Geraldo-Magela Pereira, Eduardo Nunes, Martinho-Campolina-Rebello Horta, Frank-Ferreira Silveira

**Affiliations:** 1 DDS, MS, Graduate student, Departament of Dentistry, Pontifícia Universidade Católica de Minas Gerais, Belo Horizonte, Brasil; 2 DDS, MS, PhD Professor, Department of Restorative Dentistry, Federal University of Minas Gerais, Belo Horizonte, Brazil; 3 DDS, MS, PhD, Professor, Centro Universitário Newton Paiva, Belo Horizonte, Brazil; 4 DDS, MS, PhD, Professor, Departament of Dentistry, Pontifícia Universidade Católica de Minas Gerais, Belo Horizonte, Brasil

## Abstract

**Background:**

This study aimed to evaluate changes in the enamel surface of bovine teeth after whitening with exogenous bleaching agents: 10% carbamide peroxide (group 1), 16% carbamide peroxide (group 2) and 35% hydrogen peroxide activated by a light-emitting diode (LED) (group 3). The evaluations were performed by scanning electron microscopy (SEM).

**Material and Methods:**

Ninety bovine teeth were divided into five groups (n = 18). The bleaching agents 10% and 16% carbamide peroxide were applied for eight hours a day for 14 consecutive days. The third agent, LED-activated 35% hydrogen peroxide, was used four times at seven-day intervals. Each of the four time points consisted of three applications of 10 minutes each. A 37% phosphoric acid solution and artificial saliva were used as positive and negative controls, respectively.

**Results:**

The evaluations by SEM showed changes in the enamel surfaces of the specimens. Based on the Mann-Whitney statistical test, the data showed significant differences (*p*<0.05) between groups 1 and 2 and between groups 2 and 3. However, no significant difference (*p*>0.05) was observed between groups 1 and 3.

**Conclusions:**

Based on these results, it can be concluded that bleaching agents can cause changes in the structure of tooth enamel and that these changes are related to the concentration and the duration of contact with the tooth surface.

** Key words:**Bovine teeth, carbamide peroxide, enamel, hydrogen peroxide, scanning electronic microscopy.

## Introduction

Cosmetic treatments in dentistry have become more important due to the current concept of smile aesthetics based on teeth that are white, well aligned and framed by the gums and lips. Within this context, tooth whitening takes a prominent position as one of the major wishes of patients seeking a beautiful smile.

Changes in tooth color may have extrinsic origins, when there is agglutination of pigmented foods and tobacco on the surface of the teeth, and intrinsic origin, resulting from the presence of chromogenic substances inside the enamel and dentin. The extrinsic changes can be resolved by dental prophylaxis and by alterations in the patient’s nutrition and habits. The intrinsic changes, thanks to developments in dentistry, can be solved using conservative tooth whitening techniques (1-3).

The ‘home vital bleaching’ treatment typically employs carbamide peroxide at concentrations of 10 to 16%, applied in individual trays used at night (nightguard), with eight-hour applications, or during the day, for a period of two to five weeks (1,4).

The ‘in-office’ bleaching treatment is performed using higher concentrations of peroxides, such as 35% hydrogen peroxide. Most of these peroxides are activated with the help of a light energy source, such as light emitting diodes (LEDs), providing satisfactory and fast results. LED applications are usually performed in three to four sessions at seven-day intervals (5,6).

Despite the advantages offered by vital tooth whitening, adverse side effects such as tooth sensitivity (3), changes in surface roughness (4), decreases in microhardness (7), changes in surface morphology (8) and changes in the chemical composition (9) are described in the literature.

The use of scanning electron microscopy (SEM) makes it possible to evaluate the effects of different types of bleaching agents on the tooth enamel surface, depending on composition and exposure time. Given the large number of products and techniques available for vital tooth bleaching, it is important to evaluate the effects on the tooth enamel surface, particularly in light of existing contradictory results (10).

This study aimed to verify, through SEM, the changes in the enamel surface of bovine teeth caused by bleaching agents of the same chemical composition but different concentrations, used in ‘home vital bleaching’ (10 and 16% carbamide peroxide), and a bleaching agent with a different chemical composition and higher concentration, used in ‘in-office’ bleaching (35% hydrogen peroxide) and activated by LED. The null hypothesis is that the more concentrated the gel, the greater the change caused in the tooth enamel.

## Material and Methods

-Selection and Preparation of Specimens

This study was approved by the Ethics Committee on Animal Research of the Federal University of Minas Gerais (Comitê de Ética em Experimentação Animal da Universidade Federal de Minas Gerais – CETEA), meeting the bioethics requirements.

A total of 90 freshly extracted bovine teeth were used. The teeth were cleaned with periodontal curettes (S.S. White, Petrópolis, RJ, Brazil) and running water to remove organic matter and stored in a solution of 1% sodium hypochlorite in distilled water for 24 hours. Subsequently, the bovine teeth were rinsed in running water, stored in a plastic container containing saline solution and properly identified. The teeth were sectioned in the incisal-cervical and mesiodistal directions, so that the central block, which is flatter, was obtained for testing, for a total of 90 tooth fragments. Then, the blocks were stored in labeled plastic containers containing saline solution and kept refrigerated for later inclusion.

-Inclusion of Specimens

The specimens were supported in a utility wax plate (Uraby, São Paulo, SP, Brazil), with the vestibular surface facing the inside of the wax plate and surrounded by a PVC tube of 21 mm diameter by 18 mm in height (Tubos e conexões Tigre, São Paulo, SP, Brazil), followed by the insertion of chemically activated acrylic resin (Jet Clássico, São Paulo, SP, Brazil) into the PVC tube. After the polymerization of the acrylic resin, the utility wax was removed to expose the specimens.

Subsequently, the specimens were cleaned with gauze, followed by polishing with pumice (Lenzafarm, Lenza Farmacêutica Ltda., Belo Horizonte, MG, Brazil) and a Robinson brush (KG Sorensen, Barueri, SP, Brazil). To standardize the initial roughness/smoothness of the specimens, prior surface polishing was conducted with wet sandpaper in a polisher. The specimens were washed in running water, stored in plastic containers containing saline solution and refrigerated until the first application of the bleaching agents.

Twenty-four hours prior to the application of the bleaching agents, the specimens were again cleaned with pumice and a Robinson brush, rinsed in running water and stored in artificial saliva to simulate clinical conditions.

-Distribution of Experimental Groups

The samples were randomly divided into five groups with 18 specimens each ( Table 1).

**Table 1:**
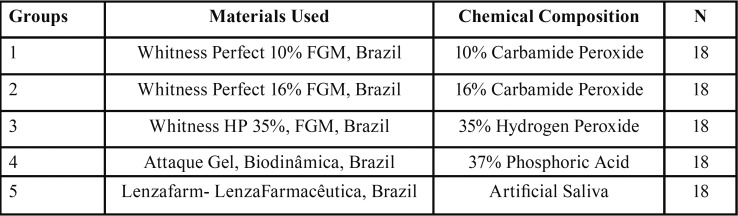
Distribution of experimental groups.

▪Group 1 - 10% Carbamide Peroxide 

The 10% carbamide peroxide (Whiteness Perfect 10% - FGM, Joinville, SC, Brazil) was applied at a thickness of approximately 1.0 mm with the tip of a syringe and spread with a brush on the surface of the specimens’ enamel. The application of 10% carbamide peroxide was carried out over a period of eight hours a day for 14 consecutive days, in a closed plastic container containing gauze soaked in saline solution. Then, the samples were washed in running water, dried with absorbent paper and stored at 37°C in the same plastic containers, containing artificial saliva, until the next application.

▪Group 2 - 16% Carbamide Peroxide

The 16% carbamide peroxide (Whiteness Perfect 16%, FGM, Joinville, SC, Brazil) was applied in the same manner as described for group 1.

▪Group 3 - 35% Hydrogen Peroxide

The application of 35% hydrogen peroxide (Whiteness HP - FGM - Joinville - SC, Brazil) and its activation with LED (Dabi Atlante - Ribeirão Preto, SP, Brazil) were performed 4 times, with seven-day intervals between each application. The ratio and mixture of the thickener and the peroxide were according to the manufacturer’s recommendations. With the aid of a spatula, the enamel surfaces of the specimens were covered in gel with a thickness of approximately 1.0 mm. After a waiting period of 1 minute, LED activation was performed for 30 seconds. At the end of this period, the gel became colorless. After 3 minutes, the gel was stirred with the aid of a brush (Atlas # 2, Esteio, RS, Brazil), followed by a second LED activation for 30 seconds. After another 3 minutes, the gel was stirred again on the surface of the enamel, followed by LED activation for 30 seconds. At the end of three activations, the 35% hydrogen peroxide gel was again stirred. After the fourth gel application, the samples were washed in running water, and the 35% hydrogen peroxide gel was gently removed with the aid of gauze and the samples dried with absorbent paper. The samples were then placed in an incubator at 37°C in plastic containers containing artificial saliva for 24 hours, as previously described. The following day, the samples were washed in running water to remove the artificial saliva, dried with absorbent paper and then placed again in an incubator at 37°C under the same conditions. These procedures were performed throughout the experimental period, and the bleaching gel was applied at seven-day intervals.

▪Group 4 - Positive Control (37% Phosphoric Acid)

The application of 37% phosphoric acid (Attaque Gel, Biodinâmica, Ibiporã, PR, Brazil), was performed only once, for 60 seconds. The application to the enamel surface was made with the tip of the syringe included in the product’s kit and spread with a brush. The samples were then washed in running water and the acid gently removed with gauze. After being placed in an incubator at 37ºC, the samples were stored in labeled plastic containers containing artificial saliva. In the remaining 13 consecutive days, the artificial saliva was renewed every 24 hours.

▪Group 5 - Negative Control (Artificial Saliva)

The specimens were immersed in artificial saliva (Lenzafarm, Lenza, Farmacêutica Ltda., Dental Division, Belo Horizonte, MG, Brazil) for 14 consecutive days. From the 1st to the 14th day, the specimens were washed in running water to remove the saliva, dried gently with gauze and placed in an incubator at 37ºC. Throughout the remaining 13 consecutive days, the artificial saliva was renewed every 24 hours.

-SEM 

All the teeth in each experimental group were analyzed by SEM (JSM-6510LV, JEOL, Japan). The specimens were dried with air jets and then stored in a desiccator with silica gel until processing for analysis. Following the drying period, the samples were fixed in aluminum stubs and taken to the metallizer for further evaluation.

Samples that were properly metallized with gold for SEM observation were selected at 1000x magnification and examined by three evaluators, with changes in the enamel surface classified as follows.

• Absent (0): unaltered surface, perikymata, presence of parallel lines, slight rugosity and development pores;

• Mild (1): irregular areas with depressions, surface presenting subtle rugosity and presence of grooves;

• Moderate (2): areas with a greater number of pores with increased diameter, presence of erosion;

• Severe (3): dissolution of enamel surface and exposure of enamel prisms.

-Statistical analysis

Differences in the enamel surface changes between the groups 1, 2 and 3 were analyzed using the Mann-Whitney test. The level of significance was set at 5%. The data were analyzed by means of GraphPad Prism software (GraphPad Software, San Diego, CA, USA).

## Results

**Figure 1:**
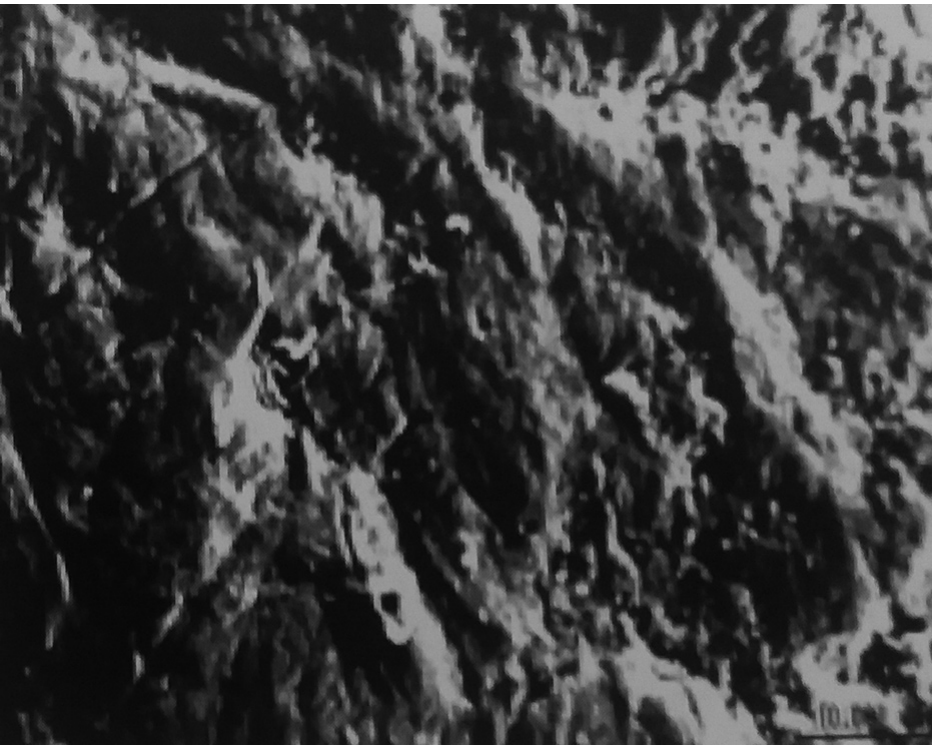
Mild changes in the enamel surface (CP 10%).

**Figure 2:**
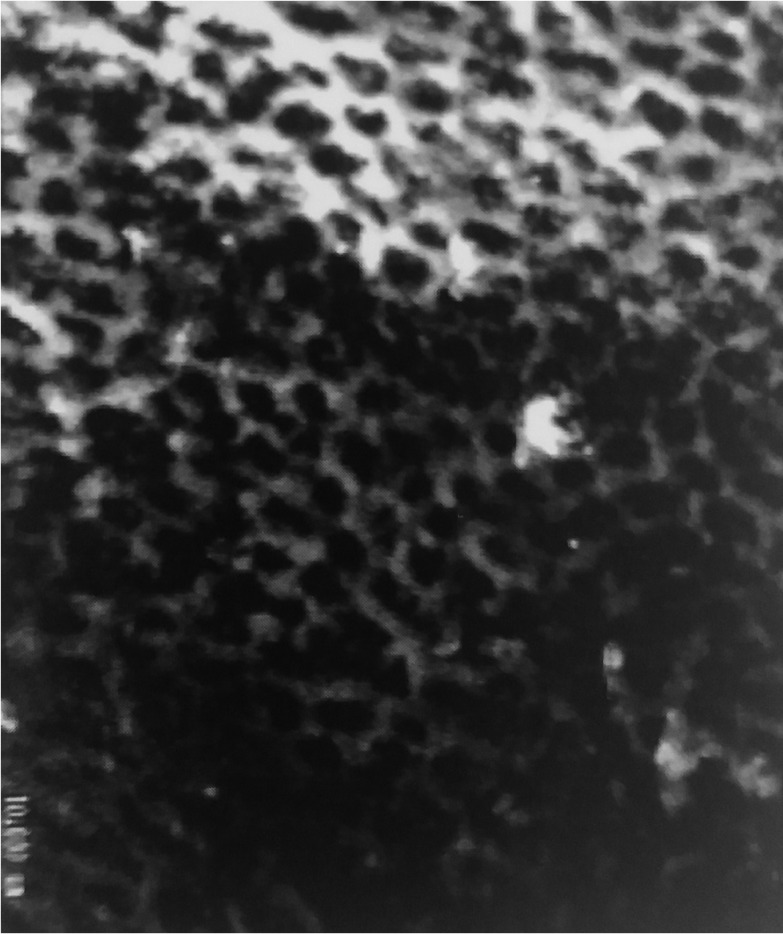
Moderate changes in the enamel surface (CP 16%).

**Figure 3:**
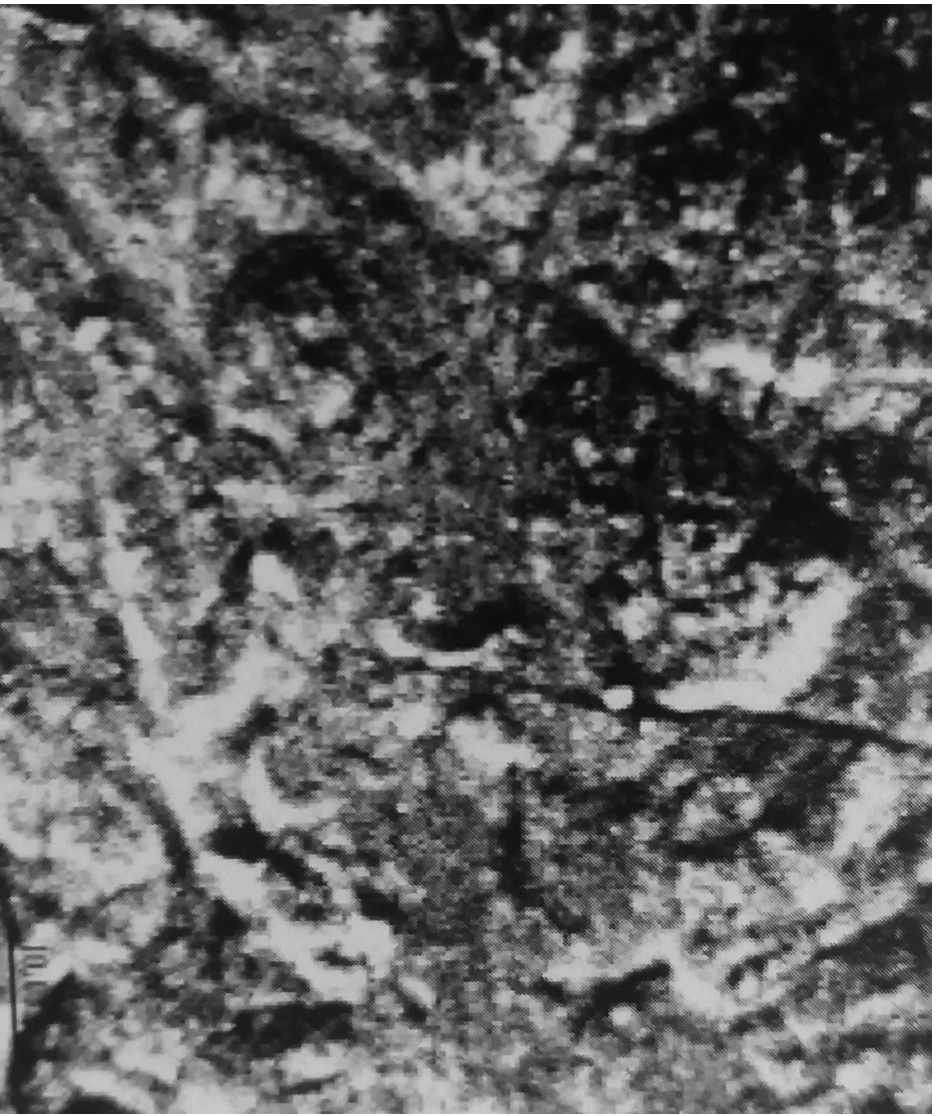
Mild changes in the enamel surface (HP 35%).

The SEM electron micrographs showed that qualitatively ( Table 2), the enamel surface of the bovine teeth showed changes due to the exposure time, composition and concentration of the whitening gel (Figs. 1-3).

**Table 2:**
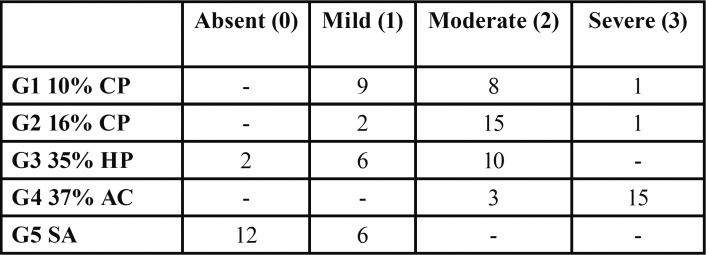
Data of the experimental groups of surface changes with different bleaching agents. Scores from 0 to 3.

The data showed significant differences (*p*<0.05) between groups 1 and 2 and between groups 2 and 3 ( Table 3). However, there was no significant difference between groups 1 and 3 (*p*>0.05).

**Table 3:**
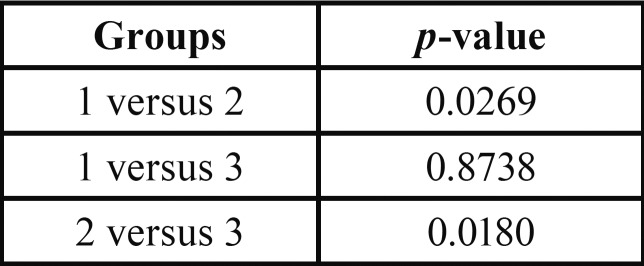
Comparative analisis among the experimental groups.

## Discussion

The choice of bovine teeth for this study was due to the ease of obtaining these specimens compared to obtaining human teeth, especially because newly extracted teeth were used. The medial region was chosen for the evaluation because it has a flat surface with approximately 1 mm thick enamel, similar to the mean thickness of human upper incisors. The physical and chemical properties of bovine teeth, such as the composition, density, depth and diameter of the enamel, and hardness, resemble human teeth (7,11).

The storage medium for specimens in *in vitro* studies can be an important factor in the observed variation of results among different studies (2). In this study, artificial saliva was used for the purpose of clinical simulation, preventing specimen demineralization (9,12), which could otherwise have interfered with the results. The use of 37% phosphoric acid as a positive control was based on the common knowledge of its potential to demineralize tooth enamel.

The use of peroxide-based bleaching agents and the development of techniques that produce a whitening effect more rapidly have been the subject of discussion in the literature because they can cause tooth sensitivity (13), tooth temperature changes (5,14), and mild changes in enamel morphology. When used at high concentrations (30-35%), hydrogen peroxide can induce chemical and morphological changes in the enamel (9,15) due to the large quantity of hydrogen ions that can bind to the calcium and phosphorus ions present in the saliva, making the oral environment subsaturated relative to the tooth structure. To maintain mineral balance, the calcium and phosphorus of the enamel are released to the saliva.

Bleaching agents should have a neutral pH, close to 7.0, to minimize adverse effects. It has been observed that in several commercial brands, the pH is acidic and not neutral as it should be; therefore, adverse side effects, such as enamel demineralization, porosity, erosion, sensitivity and gum irritation may occur with the use of these highly acidic substances. Thus, in addition to the concentration, the exposure time of these bleaching agents to the dental tissues may also influence the change in enamel micromorphology, leading to rejection of the null hypothesis. This factor explains why a high-concentration bleaching agent (group 3) produced less adverse effects than a less-concentrated bleaching agent (group 2) and statistically equal to a bleaching agent 4 times less concentrated.

This study showed that the bleaching techniques promote changes in the enamel surface to varying degrees, depending on the exposure time and the concentration of the gel used. Therefore, when choosing a tooth whitening technique, the manufacturer’s recommendations should be followed, respecting the application time according to the gel concentration selected and considering its chemical nature to understand the process.

## Conclusions

The results of this study show that bleaching agents can cause changes in the structure of the tooth enamel and that these changes are related to the concentration of the bleaching agent and its contact time with the teeth.
